# *In situ* analysis of gaseous products from PEO-based polymer electrolyte decomposition

**DOI:** 10.1039/d5sc04442a

**Published:** 2025-08-19

**Authors:** Yuan Tian, Nanbiao Pei, Jiyuan Xue, Jinzhi Wang, Haitang Zhang, Wenbin Tu, Xin Sun, Peng Zhang, Yu Qiao, Shi-Gang Sun

**Affiliations:** a State Key Laboratory of Physical Chemistry of Solid Surfaces, Department of Chemistry, College of Chemistry and Chemical Engineering, Xiamen University Xiamen 361005 PR China yuqiao@xmu.edu.cn tuwenbin@stu.xmu.edu.cn; b Discipline of Intelligent Instrument and Equipment, College of Chemistry and Chemical Engineering, Xiamen University Xiamen 361005 PR China; c College of Energy, Xiamen University Xiamen 361102 PR China pengzhang@xmu.edu.cn; d Fujian Science & Technology Innovation, Laboratory for Energy Devices (21C-Lab), Contemporary Amperex Technology Co., Limited (CATL) Ningde 352100 PR China sunx@catl.com

## Abstract

Poly(ethylene oxide) (PEO)-based polymer electrolytes have attracted considerable attention for solid-state batteries due to their excellent processability and interfacial compatibility. However, the incomplete understanding of decomposition byproducts fundamentally hinders the elucidation of degradation mechanisms and the rational design of stable interfaces. In this work, we employed online mass spectrometry and gas chromatography-mass spectrometry (GC-MS) methods to investigate the interfacial reactions between PEO-based electrolytes and activated electrodes (*e.g.* lithium metal anode and LiCoO_2_ cathode), as well as the decomposition products of PEO under both electrochemical cycling and thermal runaway conditions. In addition to permanent gases (H_2_, CO_2_, O_2_, *etc.*), we successfully tracked the dynamic evolution of several cyclic ether compounds (1,4-dioxane, ethylene oxide, dioxolane, and 2-methyl-1,3-dioxolane) with voltage-/temperature-dependence, by exploiting the efficient gas chromatographic separation capability of GC-MS for complex gaseous products. These findings provide critical insights into the dynamic degradation behavior of PEO-based electrolytes, advancing our understanding of their decomposition pathways under varying operational conditions and establishing a material design framework for the rational development of next-generation polymer electrolytes.

## Abstract 2

Unveiling the voltage- and temperature-dependent evolution of gaseous products from PEO-based electrolytes using online MS and GC-MS, elucidating thermally and electrochemically triggered decomposition mechanisms and intermediate evolution.

## Graphical abstract

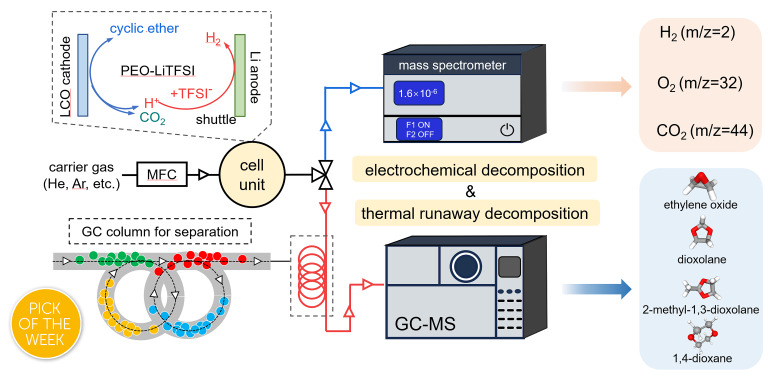

## Introduction

The pursuit of high-energy-density rechargeable batteries has driven the rapid development of electrode materials, but electrode–electrolyte interfacial challenges have severely limited battery applications.^1–3^ Although high-voltage cathodes (*e.g.*, LiCoO_2_, Li(Ni_*x*_Co_*y*_Mn_1−*x*−*y*_)O_2_, and LiNi_0.5_Mn_1.5_O_4_) and the lithium metal anode can significantly increase energy density,^4–6^ their parasitic side reactions with liquid electrolytes lead to cycling stability degradation and safety hazards. Poly(ethylene oxide) (PEO)-based solid-state electrolytes are considered potential solutions due to their interfacial compatibility and processing advantages. However, their applications are limited by bottlenecks including a narrow electrochemical window, low room-temperature ionic conductivity, poorly understood decomposition mechanisms and interfacial parasitic reactions.^7–11^

The interfacial reaction mechanisms and dynamic degradation pathways between PEO-based electrolytes and electrodes remain poorly understood, primarily due to technical limitations in characterizing solid-state electrolyte interfaces. In conventional liquid electrolyte systems, interfacial parasitic reactions exhibit a certain degree of self-limiting behaviour, which facilitates the formation of structurally well-defined cathode electrolyte interphases (CEIs) and solid electrolyte interphases (SEIs). These interphases play a crucial role in maintaining the stability of lithium batteries (LBs).^12–17^ The compositional evolution of such interphases can be effectively characterized using advanced analytical techniques, such as time-of-flight secondary ion mass spectrometry and X-ray photoelectron spectroscopy.^18–20^ However, in PEO-based polymer electrolyte systems, the inseparable nature of the electrode/electrolyte interface poses fundamental limitations for post-mortem analysis. More critically, the dynamic evolution of decomposition products at both cathode and anode interfaces has not been systematically analysed during prolonged cycling. This incomplete identification of decomposition products directly hinders mechanistic understanding of interfacial degradation processes, thereby constraining rational design strategies for PEO-based polymer electrolytes.

A pioneer study characterized the gaseous evolution of PEO-based polymer electrolytes under high-voltage conditions using differential electrochemical mass spectrometry (DEMS).^21^ This representative work revealed that ethylene oxide (EO) chain segments and lithium salts undergo significant electrochemical degradation. Specifically, when the charging voltage exceeds 4.5 V (*vs.* Li^+^/Li), PEO undergoes exacerbated decomposition accompanied by gas evolution (H_2_, CO_2_, O_2_, C_2_H_4_, *etc.*), leading to substantial capacity fading of the LiCoO_2_‖PEO‖Li cell. However, chemical identification and speciation of PEO decomposition products remain incomplete. In another word, the cleavage of long PEO chains not only produces the aforementioned small-molecule gaseous products that have been identified, but its decomposition process also inevitably involves complex radical reactions and organic transformation pathways, likely leading to the formation of intermediate organic products such as alcohols, ethers, or esters during the decomposition process. At elevated temperatures such as 60 °C, these intermediate products may also be released in gaseous form. However, systematic studies on the types of these intermediates and their reaction mechanisms are still lacking. This knowledge gap hinders a clear understanding of the decomposition mechanism and pathways of PEO, and consequently limits our ability to perform targeted modifications to improve its interfacial stability. In addition to electrochemical decomposition, thermal runaway represents another critical practical scenario in battery applications, yet systematic investigations into the gas evolution behaviour of PEO-based electrolytes under such conditions remain scarce. Accordingly, the development of precise *in situ* analytical techniques is critically needed to unravel the degradation mechanisms occurring at solid–solid interfaces. Among them, *in situ* gas analysis has proven to be a particularly effective strategy for identifying gaseous byproducts and elucidating interfacial reaction pathways, thereby providing essential guidance for the concurrent optimization of cathode and electrolyte materials.^22,23^

In this study, we employed online mass spectrometry (MS) coupled with a custom-built online gas chromatography–mass spectrometry (GC-MS) system to systematically analyse the gaseous decomposition products generated from PEO-based electrolytes under electrochemical cycling and thermal runaway conditions. The experimental results revealed dynamic evolution patterns dominated by inorganic gases (H_2_, CO_2_, and O_2_) and cyclic ether derivatives. Specifically, we elucidated the H_2_ evolution mechanism at the PEO/lithium metal anode interface. Moreover, we also established a deeper understanding of oxygen-containing organic decomposition products (*e.g.* ethers) formed under high-voltage operation and thermal abuse conditions. These findings establish a theoretical foundation for elucidating the decomposition pathways of PEO-based electrolytes and provide valuable guidance for the rational design of next-generation PEO electrolytes with wider electrochemical stability windows, enhanced safety, and improved interfacial stability in advanced battery systems.

## Results and discussion

### Dehydrogenation of PEO on lithium metal

In the preparation of PEO-based polymer electrolytes *via* the solution casting method, solvents such as acetonitrile (ACN) or *N*,*N*-dimethylformamide (DMF) effectively dissolve PEO and lithium bis((trifluoromethyl)sulfonyl)azanide (LiTFSI).^24,25^ Subsequent drying processes remove these solvents to form solid electrolyte membranes, where the drying duration critically influences the residual solvent content within the membranes. For lithium metal batteries, PEO electrolytes require the formation of a stable SEI at the anode interface to ensure cycling stability. However, the influence of residual solvents on SEI composition and their parasitic reaction mechanisms with lithium metal remain unresolved.

**Fig. 1:**
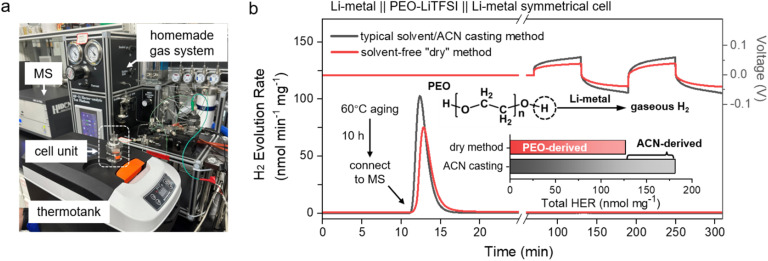
(a) The home-made online-MS test system. (b) The OEMS analysis of H_2_ evolution behaviour during high-temperature stabilization and galvanostatic cycling in Li-metal‖PEO–LiTFSI‖Li symmetric cells with different electrolyte membrane preparation methods. After cell assembly, the electrode/electrolyte interfaces were stabilized at 60 °C for 10 h, followed by online mass spectrometry (MS) monitoring of the H_2_ evolution rate and the voltage profiles during galvanostatic cycling at a constant current of 0.1 mA. The bottom inset bar chart shows the total H_2_ evolution (normalized to polymer mass, nmol mg^−1^), separated into contributions derived from intrinsic PEO decomposition (PEO-derived) and residual solvent/acetonitrile decomposition (ACN-derived).

In this section, online electrochemical mass spectrometry (OEMS) was employed to *in situ* monitor gaseous products generated at the electrode–electrolyte interface. Fig. 1a presents the schematic of our homemade gas analysis system, in which the battery is assembled within a custom-designed cell unit and placed in a thermostatic chamber for controlled heating. A PEO-based lithium symmetric cell (Li‖PEO–LiTFSI‖Li) was first thermally stabilized at 60 °C for 10 hours and subsequently connected to a mass spectrometry system for gas evolution analysis. Fig. 1b displays the voltage profiles and corresponding H_2_ evolution rates of symmetric cells assembled with PEO-based polymer electrolyte membranes prepared by either solvent casting or dry processing, during both interfacial stabilization and subsequent charge–discharge cycling at 60 °C. The quantities of H_2_ released during the thermal stabilization phase and the electrochemical cycling phase were quantitatively determined. It is evident that H_2_ evolution occurs during the high-temperature stabilization stage, even in the absence of electrochemical cycling, for both types of PEO-based electrolytes. Notably, the symmetric cell using the solvent-cast membrane exhibits a significantly higher H_2_ release of 181.58 nmol mg^−1^, compared to 126.96 nmol mg^−1^ for the cell with the dry-processed membrane. These results indicate that PEO undergoes spontaneous chemical reduction in contact with lithium metal at elevated temperature, leading to H_2_ evolution. This process is primarily attributed to the reaction between terminal hydroxyl groups (–OH) in the PEO chains and lithium metal, as represented by the following equation:HO–(CH_2_CH_2_O)_*n*_–H + Li → LiO–(CH_2_CH_2_O)_*n*_–H + 0.5H_2_.

In addition, residual acetonitrile may exacerbate H_2_ generation, either by directly reacting with lithium metal under insufficient drying conditions or by facilitating the dehydrogenation kinetics of PEO terminal groups through localized proton-conducting pathways formed by trace solvent residues. Notably, no H_2_ evolution was detected by OEMS during subsequent charge–discharge cycles (Fig. 1b), indicating that the solid electrolyte interphase (SEI) formed during the high-temperature stabilization process effectively passivates the interface and prevents further direct contact between PEO and lithium metal, thereby suppressing side reactions during cycling.

This result provides mechanistic insights into the development of PEO-based polymer electrolytes with improved compatibility toward lithium metal. To mitigate the intrinsic reductive dehydrogenation at the PEO/lithium interface, future research should prioritize terminal group engineering strategies, such as etherification of hydroxyl groups to reduce their chemical dehydrogenation reactivity.^26,27^ Moreover, replacing high-polarity solvents (*e.g.* ACN and DMF) with low-polarity alternatives (*e.g.* propylene carbonate) may effectively suppress solvent-induced parasitic reaction pathways.^28^

### Online electrochemical MS/GC-MS analysis of high-voltage decomposition of PEO

**Fig. 2:**
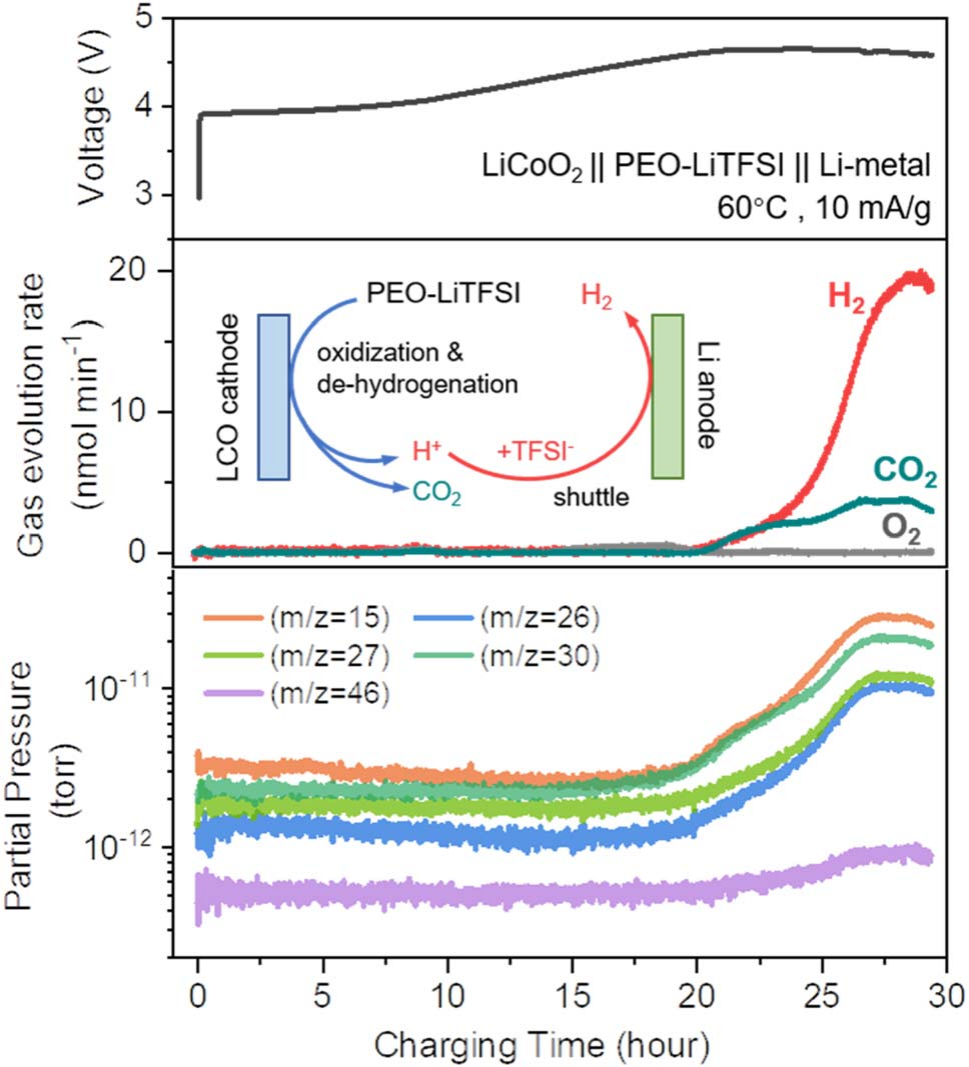
Electrochemical charging profile and corresponding OEMS analysis of a LiCoO_2_‖PEO–LiTFSI‖Li-metal cell. The top panel shows the voltage profile during galvanostatic charging at 60 °C with a constant current of 10 mA g^−1^. The middle panel displays the evolution rates of H_2_ (red), CO_2_ (teal), and O_2_ (gray) as a function of charging time, expressed in nmol min^−1^ mg^−1^ (normalized to polymer mass). A significant increase in CO_2_ and H_2_ evolution is observed when the voltage approaches 4.6 V (*vs.* Li^+^/Li), while O_2_ remains undetectable throughout the entire process. The inset illustrates the proposed interfacial decomposition and shuttle mechanism within the PEO–LiTFSI electrolyte: under high-voltage conditions, PEO undergoes oxidative decomposition at the LiCoO_2_ interface to release CO_2_; meanwhile, dehydrogenation generates H^+^, which protonates TFSI^−^ to form HTFSI. Then, the HTFSI shuttles to the Li-metal anode and reacts with Li to produce H_2_. The bottom panel presents the logarithmic-scale partial pressures (torr) of selected mass-to-charge (*m*/*z*) signals over the charging process. All traces remain near baseline levels during early stages, but begin to rise prior to 4.6 V (*vs.* Li^+^/Li), indicating that unidentified gaseous components would accumulate before the onset of bulk CO_2_ and H_2_ evolution.

Furthermore, to investigate the oxidative decomposition mechanisms of PEO-based polymer electrolytes under high-voltage conditions, we focus on the LiCoO_2_ (LCO) cathode/PEO-based electrolyte interface from the perspective of gas evolution analysis and deliberately perform OEMS measurements on an electrochemical cell with a LiCoO_2_‖PEO–LiTFSI‖Li configuration, employing solvent-cast PEO-based polymer electrolyte membranes. The dynamic evolution of various permanent gases is monitored during the overcharging process. As shown in the top panel of Fig. 2, the time-voltage profile is recorded in parallel with the gas evolution rates (middle panel, Fig. 2) of different gas compounds. Remarkably, no detectable O_2_ (*m*/*z* = 32) evolution is observed throughout the charging process, even when charging to 4.6 V (*vs.* Li^+^/Li). Such a phenomenon stands in sharp contrast to our previous findings with LCO‖Li half-cells using liquid electrolytes, where significant O_2_ evolution was detected under comparable conditions.^29^ On the one hand, we propose that the capacity contribution observed under high-voltage conditions primarily arises from electrolyte oxidation, rather than from the delithiation process of the LCO cathode. In the PEO-based solid-state battery system, the delithiation capacity of the LCO cathode does not reach the critical threshold for lattice oxygen release, resulting in the absence of detectable O_2_ evolution. On the other hand, even if trace amounts of O_2_ were released, they would likely undergo rapid reactions with the PEO-based polymer electrolyte and be completely consumed.^30^ Subsequently, pronounced generation of both H_2_ (*m*/*z* = 2) and CO_2_ (*m*/*z* = 44) is distinctly observed when charged above 4.6 V (*vs.* Li^+^/Li). This behaviour can be explained by the following reaction pathway: under high-voltage conditions, oxidative dehydrogenation occurs at both hydroxyl (–OH) terminals and ether (–O–) linkages in PEO chains, producing protons (H^+^) that subsequently react with TFSI^−^ anions to form the highly acidic HTFSI intermediate. Thus, the HTFSI generated at the LCO cathode interface may diffuse through the electrolyte to the Li metal anode interface, where it undergoes reduction (HTFSI + Li → LiTFSI + 0.5H_2_), resulting in H_2_ evolution.^21^ Concurrently, the PEO matrix undergoes radical-initiated chain scission under high-voltage oxidative conditions, ultimately generating CO_2_ through a series of complex reactions.

Notably, prior to reaching 4.6 V (*vs.* Li^+^/Li), gradual increases in signals at *m*/*z* = 15, 26, 27, 30, and 46 were observed (bottom section, Fig. 2). Previous reports have conventionally assigned these *m*/*z* values to common volatile species, including methane (CH_4_, *m*/*z* = 15), acetylene (C_2_H_2_, *m*/*z* = 26), ethylene (C_2_H_4_, *m*/*z* = 27), ethane (C_2_H_6_, *m*/*z* = 30), and ethanol (C_2_H_5_OH, *m*/*z* = 46), based on characteristic fragmentation patterns. However, the inherent limitations of quadrupole mass spectrometry with electron impact (EI) ionization may lead to inaccurate product identification through this single m/z-based qualitative approach. While quadrupole systems enhance sensitivity for specific *m*/*z* values through fixed radiofrequency (RF) and direct current (DC) voltage combinations, they sacrifice the detection range of *m*/*z*. Given that PEO consists of repeating (–CH_2_–CH_2_–O–) units and primarily decomposes *via* ether bond cleavage, it is likely to generate radical intermediates that can undergo complex secondary reactions to form unstable organic species such as alcohols, ethers, and esters. While these intermediates may ultimately oxidize into short-chain volatile products, their fragmentation under EI conditions produces *m*/*z* signals that overlap with those of simpler molecules, rendering accurate identification of the intermediate species highly challenging. For instance, methyl-containing intermediates may generate CH_3_^+^ fragments (*m*/*z* = 15) that could be misattributed to CH_4_ due to the mass spectrometry signal overlap.

**Fig. 3:**
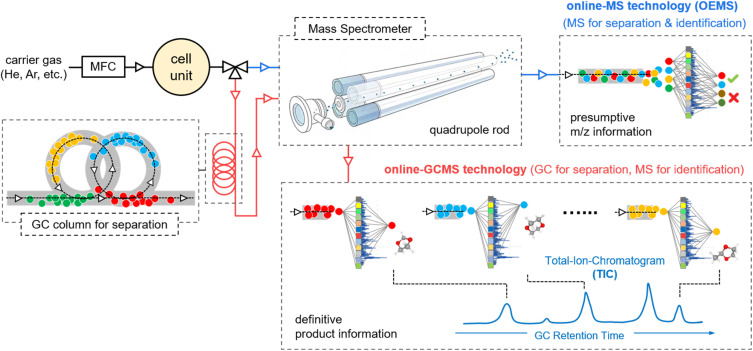
Schematic illustration comparing the qualitative/quantitative principles of OEMS and online GC-MS. OEMS enables real-time detection of gas products by monitoring pre-selected ion fragments (*m*/*z*) through a quadrupole MS analyser. However, the use of electron ionization (EI) typically causes extensive fragmentation, which hampers the reliable identification of complex organic species. In addition, OEMS is inherently limited to a narrow set of predefined *m*/*z* values, making it more suitable for targeted analysis of known gases rather than for the discovery of unknown compounds. In contrast, online GC-MS separates volatile decomposition products *via* a GC column prior to MS analysis. This separation minimizes spectral overlap and preserves the molecular identity of analytes. By combining retention time information with characteristic fragmentation patterns, and comparing them against a standard MS database, GC-MS allows accurate and unambiguous identification of a wide range of complex organic compounds, including previously unknown species evolved during electrolyte decomposition.

To enable precise qualitative/quantitative analysis of oxidative decomposition by-products in PEO-based polymer electrolytes and elucidate the underlying reaction mechanisms, we employed a custom-designed online electrochemical GC-MS system developed in our previous work.^31^ Compared to conventional OEMS analysis that directly couples electrochemical cells with mass spectrometers, this system integrates gas chromatography (GC) as a front-end separation module prior to mass spectrometric detection (Fig. 3). The decomposition products undergo differential migration through the GC column, exhibiting distinct retention times governed by their adsorption affinities and solubility parameters in the stationary phase. Such chromatographic separation enables sequential elution of analytes, effectively resolving the typically signal overlap in conventional OEMS spectra.

**Fig. 4:**
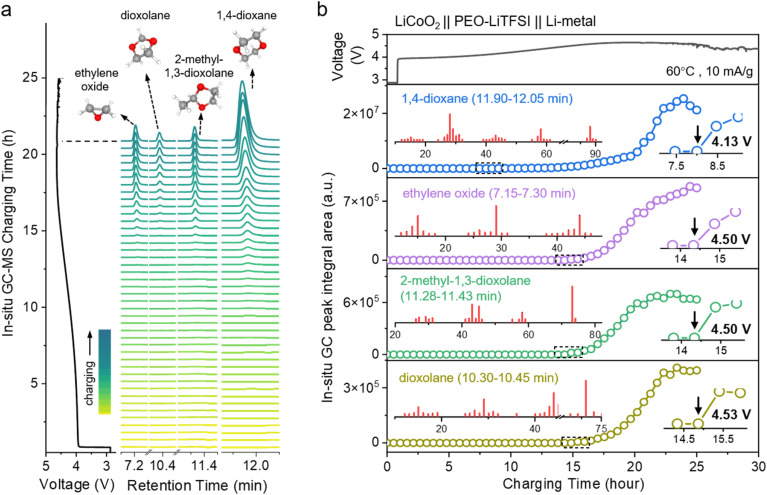
Online electrochemical GC-MS characterization of cyclic ether intermediates during high-voltage charging of a LiCoO_2_‖PEO–LiTFSI‖Li-metal cell. (a) Total ion chromatograms (TICs) recorded using a custom-built online GC-MS system during overcharging process, showing four distinct peaks identified—*via* database matching—as ethylene oxide (7.15–7.30 min), dioxolane (10.30–10.45 min), 2-methyl-1,3-dioxolane (11.28–11.43 min), and 1,4-dioxane (11.90–12.05 min). (b) Evolution of baseline-corrected TIC peak areas for each cyclic ether species as a function of cell voltage and time during galvanostatic overcharging, plotted alongside the corresponding voltage profile (top). 1,4-Dioxane is first detected at 4.13 V (*vs.* Li^+^/Li), while ethylene oxide, dioxolane, and 2-methyl-1,3-dioxolane begin to appear above 4.50 V (*vs.* Li^+^/Li), indicating a staged formation of volatile intermediates as the charging voltage increases.

Online electrochemical GC-MS analysis was performed on a LiCoO_2_‖PEO–LiTFSI‖Li half-cell under overcharge conditions, with the acquired total ion chromatograms (TICs) shown in Fig. 4a. Each GC peak corresponded to a distinct volatile decomposition species, with four structurally distinct cyclic ether derivatives being conclusively identified through matching with the database. The compounds eluted sequentially in order of ascending retention times as follows: ethylene oxide (7.15–7.30 min), dioxolane (10.30–10.45 min), 2-methyl-1,3-dioxolane (11.28–11.43 min), and 1,4-dioxane (11.90–12.05 min). Specifically, based on the time-voltage curves recorded during battery testing coupled with quantitative analysis of gaseous decomposition products *via* GC-MS peak integration (Fig. 4b), the initial appearance of 1,4-dioxane was detected at 4.13 V (*vs.* Li^+^/Li), whereas the formation of ethylene oxide, dioxolane, and 2-methyl-1,3-dioxolane commenced upon exceeding the critical voltage threshold of 4.50 V (*vs.* Li^+^/Li). The formation kinetics of all four cyclic ether species accelerated with increasing voltage, exhibiting a sharp increase in production rates within the 4.5–4.8 V (*vs.* Li^+^/Li) range, which aligns precisely with voltage fluctuations associated with the oxidative decomposition of the PEO-based polymer electrolyte. These ether derivatives underwent sequential oxidative degradation during subsequent decomposition processes, culminating in complete mineralization into gaseous CO_2_. This synchronization indicates that the PEO backbone undergoes irreversible and severe decomposition at 4.5 V (*vs.* Li^+^/Li). The massive cleavage of ether bonds not only triggers an explosive accumulation of gaseous byproducts but also induces structural collapse of the ion-transport network at the electrode/electrolyte interface, ultimately resulting in as rapid capacity decay in the battery.

The online electrochemical GC-MS system effectively captured the stepwise evolution of characteristic cyclic ether derivatives originating from the cleavage–recombination of ether bonds (–O–) within PEO-based polymer electrolytes during voltage ramping. The distinct onset potentials of these species reveal voltage-dependent generation pathways closely associated with the increasing oxidative potential. Based on GC-MS detection of 1,4-dioxane formation starting at 4.13 V (*vs.* Li^+^/Li), this finding challenges the conventional understanding based on traditional OEMS measurements, where the release of inorganic gases such as CO_2_ (typically observed around 4.5 V (*vs.* Li^+^/Li)) was considered the onset indicator of PEO decomposition and gas generation. For the first time, we recognize that the decomposition of PEO and the associated gas evolution may begin at a significantly lower potential. These results not only highlight the limitations of conventional OEMS in resolving the complex degradation mechanisms of polymer electrolytes, but also demonstrate the utility of GC-MS as a powerful analytical tool for accurately identifying degradation pathways. Furthermore, this approach provides a methodological framework for the rational design of high-stability electrolyte systems, offering critical insights for the precise mapping of decomposition processes and the strategic optimization of molecular engineering at the polymer chain ends.

### Online pyrolysis MS/GC-MS analysis of thermal runaway decomposition of PEO

**Fig. 5:**
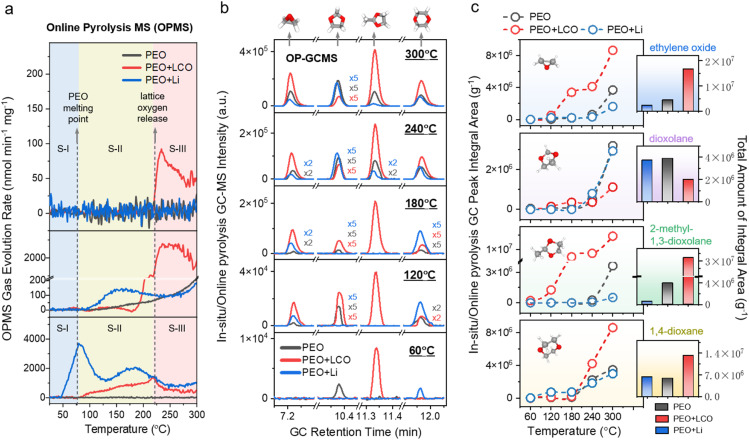
Thermal runaway gassing behaviour of PEO-based electrolytes: OP-MS and OP-GC-MS analysis of pure PEO, PEO + Li, and PEO + LCO systems. (a) Temperature-dependent gas evolution rate profiles for H_2_, CO_2_, and O_2_, obtained by OP-MS during a programmed heating ramp in three sample configurations: pure PEO, PEO + Li metal, and PEO + LiCoO_2_. The thermal decomposition process is roughly divided into three distinct stages based on critical temperature thresholds: stage I (25–80 °C), stage II (80–218 °C), and stage III (≥218 °C). (b) Temperature-dependent formation curves of four representative cyclic ether intermediates—ethylene oxide, dioxolane, 2-methyl-1,3-dioxolane, and 1,4-dioxane—during thermal decomposition of pure PEO, PEO + Li, and PEO + LCO systems, as determined by OP-GC-MS analysis. (c) Temperature-dependent formation and cumulative yields of cyclic ether intermediates in pure PEO, PEO + Li, and PEO + LCO systems. The left panel (point-line plot) shows the temperature-dependent yields of ethylene oxide, dioxolane, 2-methyl-1,3-dioxolane, and 1,4-dioxane during a programmed heating ramp, as obtained from OP-GC-MS analysis. Each point represents a baseline-corrected TIC peak area at a selected temperature, reflecting the thermal formation kinetics of each intermediate. The right inset (bar chart) compares the cumulative yields of the three samples—expressed as baseline-corrected TIC peak areas—of the four cyclic ethers at the final temperature of the heating process.

Thermal runaway not only causes a rapid rise in internal temperature and pressure within lithium batteries but may also trigger violent combustion or even explosion, posing severe safety hazards. Therefore, a comprehensive understanding of the material transformations and gas evolution mechanisms during thermal runaway is essential for the rational design of safer energy storage systems.^32^ To systematically elucidate the thermal degradation behaviour and the temperature-dependent evolution of decomposition products in PEO-based polymer electrolytes under thermal runaway conditions, thermal decomposition analyses were conducted using online pyrolysis mass spectrometry (OP-MS) and online pyrolysis gas chromatography-mass spectrometry (OP-GCMS). These investigations were performed on both pure PEO systems and composite configurations incorporating either lithium metal anodes (PEO + Li) or LCO cathodes (PEO + LCO). Fig. 5a compiles the OP-MS data for the three aforementioned systems, with a particular focus on comparing the temperature-dependent evolution of H_2_, CO_2_, and O_2_ generation rates across different composite electrode configurations. The results reveal that PEO-based polymer electrolytes exhibit distinct thermal decomposition behaviours depending on the electrode composition. This process can be broadly divided into three stages, demarcated by two critical thermal thresholds: the melting point of PEO (80 °C) and the onset temperature for lattice oxygen release from the LCO cathode (218 °C).

(i) Stage I (25–80 °C): during the initial heating stage, H_2_ evolution was detected exclusively in the PEO + Li system, beginning in the mid-range of this temperature window. No gas generation was observed in either the pure PEO or PEO + LCO systems. This early H_2_ evolution in the PEO + Li system is attributed to intrinsic reduction reactions between lithium metal and the polymer, particularly involving terminal hydroxyl groups.

(ii) Stage II (80–218 °C): as the temperature increases past the melting point of PEO, the H_2_ evolution rate in the PEO + Li system initially decreases, likely due to the melting of PEO, which enhances polymer fluidity, improves interfacial wettability, and promotes redistribution of the passivation layer over the lithium surface, thereby temporarily suppressing H_2_ release. Around 130 °C, a resurgence in H_2_ generation is observed, possibly due to partial degradation of the passivation layer or deeper infiltration of molten polymer that exposes fresh lithium surfaces. Beyond 180 °C, H_2_ evolution declines again as PEO undergoes further carbonization and crosslinking, forming thermally stable structures that inhibit interfacial reactions with lithium. By contrast, the PEO + LCO system begins to generate H_2_ in stage II through Co^3+/4+^-catalyzed dehydrogenation of the polymer. At the same time, CO_2_ evolution commences in the pure PEO system and increases steadily with temperature. The PEO + Li system shows significantly higher CO_2_ generation rates compared to pure PEO, which is attributed to lithium-promoted interfacial redox reactions with oxygen-containing decomposition intermediates, thereby accelerating polymer breakdown and enhancing CO_2_ release.

(iii) Stage III (≥218 °C): at this stage, thermal destabilization of LCO leads to the release of lattice oxygen, a process unique to the PEO + LCO system. This triggers a sharp increase in CO_2_ evolution—reaching nearly ten times the level observed in the pure PEO system. The surge in CO_2_ release is attributed to the synergistic effects of Co^3+/4+^-catalyzed decomposition of the polymer and secondary oxidation of volatile intermediates by the liberated lattice oxygen. Simultaneously, H_2_ evolution is strongly suppressed, likely due to oxidative consumption *via* reaction with reactive oxygen species (*e.g.*, 2H_2_ + O_2_ → 2H_2_O). These findings highlight the critical role of cathode-derived oxygen species in modulating gas-phase reaction pathways and accelerating thermal runaway in PEO-based electrolytes.

OP-GCMS analysis revealed that the four cyclic ether compounds generated during high-voltage oxidative decomposition were also detected during thermal runaway, each displaying distinct temperature-dependent formation behaviours. Although the onset temperatures for the formation of these cyclic ethers differed among the pure PEO, PEO + Li, and PEO + LCO systems, their production rates increased steadily with rising temperature across all cases (Fig. 5b). Notable changes in formation kinetics were observed when PEO was combined with either lithium metal or LCO, reflecting the influence of electrode composition on decomposition pathways. In particular, the PEO + LCO system exhibited a significantly higher total yield for three of the four cyclic ethers, excluding dioxolane, compared to pure PEO. This enhancement suggests that LCO promotes PEO degradation through catalytic processes while simultaneously suppressing the radical pathways responsible for dioxolane formation (Fig. 5c). Additionally, the initial appearance of 2-methyl-1,3-dioxolane in this system occurred at a notably lower temperature of 60 °C, nearly 180 °C below that observed in the pure PEO and PEO + Li systems. Both 2-methyl-1,3-dioxolane and ethylene oxide began forming rapidly from 120 °C onward, with markedly elevated production rates relative to the other systems (Fig. 5b). These observations indicate that reactive species such as Co^4+^ ions or lattice oxygen from LCO likely catalyse the conversion of thermally labile polymer fragments into oxygen-containing cyclic products. By contrast, the PEO + Li system showed diminished yields of ethylene oxide and 2-methyl-1,3-dioxolane at temperatures above 240 °C when compared to pure PEO. This reduction suggests that these volatile intermediates undergo further consumption through secondary reactions with lithium metal, possibly involving nucleophilic substitution or radical quenching mechanisms that become prominent at elevated temperatures.

These findings collectively demonstrate that the thermal decomposition behaviour of PEO-based polymer electrolytes is significantly influenced by the nature of the electrode materials. LCO promotes extensive degradation of PEO through catalytic pathways, accelerating the generation of volatile decomposition products, while metallic lithium exerts a scavenging effect by chemically reacting with and thereby suppressing the accumulation of specific cyclic ethers. This electrode-dependent modulation of decomposition not only shapes the dynamic evolution profiles of gaseous products during thermal runaway, but also reveals critical differences in thermal stability among the resulting intermediates. In particular, cyclic ether compounds formed during decomposition exhibit much lower thermal stability than the bulk polymer, are prone to forming explosive mixtures with air, and possess anesthetic properties upon inhalation, all of which considerably elevate safety risks under both standard operating conditions and failure scenarios. These insights highlight the urgent need for molecular design strategies that can inhibit ether bond cleavage and guide the reaction pathway toward the formation of thermally stable, crosslinked structures, thereby enhancing the intrinsic safety of PEO-based electrolytes.

## Conclusions

In summary, we systematically integrated *in situ* mass spectrometry (MS) with gas chromatography-mass spectrometry (GC-MS) to monitor the gas evolution behaviour of PEO-based polymer electrolytes in LiCoO_2_‖PEO–LiTFSI‖Li cells under both electrochemical and thermal runaway conditions. This comprehensive characterization platform enabled the detection of both permanent gases (H_2_, O_2_, CO_2_, *etc.*) and volatile organic compounds (such as 1,4-dioxane, ethylene oxide, dioxolane, and 2-methyl-1,3-dioxolane). The decomposition behaviour of PEO was re-examined using this system, yielding two novel insights. First, concerning the onset potential for electrochemical decomposition into volatile species, conventional OEMS studies have typically relied on CO_2_ evolution as a marker of PEO degradation. However, as CO_2_ is generally a terminal oxidation product, this may underestimate the true onset of decomposition. In contrast, our electrochemical GC-MS analysis detected the emergence of 1,4-dioxane at 4.13 V (*vs.* Li^+^/Li), suggesting that this intermediate may serve as a more sensitive and earlier indicator of the onset of PEO oxidation. Second, with respect to the decomposition pathway, the concurrent detection of CO_2_ and lower-molecular-weight cyclic ethers indicates that the degradation of PEO under these conditions is often incomplete, proceeding through multiple intermediate stages rather than terminating solely in small gaseous molecules. These intermediates possess lower thermal and electrochemical stability, underscoring the need for targeted material modifications that facilitate their full degradation. Additionally, mechanistic analyses under different failure modes reveal that (1) PEO undergoes spontaneous reductive dehydrogenation upon coming in contact with lithium metal, releasing H_2_, a process further exacerbated by residual solvents; however, this can be suppressed by stable SEI formation during pre-aging. (2) At high voltages, ether bond cleavage in the PEO backbone produces several volatile cyclic intermediates, while PEO oxidative dehydrogenation promotes protonation of TFSI^−^ anions to form HTFSI, which migrates to the anode and induces further H_2_ evolution, aggravating interfacial instability. (3) Under thermal runaway, the LCO cathode accelerates PEO decomposition *via* both lattice oxygen release and catalytic effects of high-valence cobalt (Co^3+/4+^), while the lithium anode not only reacts directly with PEO to produce H_2_ but also further reacts with ether intermediates released during PEO pyrolysis.

Overall, this study establishes a comprehensive mechanistic framework for understanding the dynamic degradation pathways of PEO-based polymer electrolytes. The adoption of an online GC-MS platform overcomes the resolution limitations of conventional MS techniques, providing a robust methodology for deciphering multi-step decomposition processes. These insights offer critical guidance for the rational design of next-generation solid-state electrolytes with improved high-voltage resilience and thermal safety.

## Author contributions

Y. T. and N. P. contributed equally to this work. Y. T. and Y. Q. contributed to the design of the research and performed the experimental data analysis. N. P. conducted the preparation of polymer electrolytes. W. T., P. Z. and Y. Q. supervised the work. All authors discussed the results and co-wrote and commented on the manuscript.

## Conflicts of interest

There are no conflicts to declare.

## Supplementary Material

SC-OLF-D5SC04442A-s001

## Data Availability

The data that support the findings of this study are available from the corresponding author upon reasonable request. The detailed experimental procedures for battery assembly and MS/GC-MS analyses are provided in the supporting information. See DOI: https://doi.org/10.1039/d5sc04442a.
