# Supplementary material for: In situ analysis of gaseous products from PEO-based polymer electrolyte decomposition
Source: Chem Sci. 2025 Aug 19;16(39):18126–34. doi: 10.1039/d5sc04442a (PMC12421431; doi:10.1039/d5sc04442a)
Supplement: SC-016-D5SC04442A-s001 [file SC-016-D5SC04442A-s001.pdf]

Supplementary Information (SI) for Chemical Science.
This journal is © The Royal Society of Chemistry 2025

Supporting Information

In situ analysis of gaseous products from PEO-based polymer electrolyte decomposition

Yuan Tian,[a,b] Nanbiao Pei, [a] Jiyuan Xue, [a,b] Jinzhi Wang, [d] Haitang Zhang, [a] Wenbin Tu, *[a,b] Xin Sun, *[d] Peng Zhang, * [c] Yu Qiao, *[a] and Shi-Gang Sun[a]

Author affiliations

[a] State Key Laboratory of Physical Chemistry of Solid Surfaces, Department of Chemistry, College of Chemistry and Chemical Engineering, Xiamen University, Xiamen, 361005, PR China.

[b] Discipline of Intelligent Instrument and Equipment, College of Chemistry and Chemical Engineering, Xiamen University, Xiamen, 361005, PR China

[c] College of Energy, Xiamen University, Xiamen 361102, P.R. China.

[d] Fujian Science & Technology Innovation, Laboratory for Energy Devices (21C-Lab), Contemporary Amperex Technology Co., Limited (CATL), Ningde, 352100, PR China.

Experimental Section / Methods

1. Materials and Chemicals

Poly(ethylene oxide) (PEO) was purchased from Shanghai Aladdin Biochemical Technology Co., Ltd. (China). Lithium bis ((trifluoromethyl) sulfonyl) azanide (LiTFSI) was purchased from Zhangjiagang Guotai-Huarong New Chemical Materials Co., Ltd. (China).

0.36 g of LiTFSI powder was dissolved in 15 mL of acetonitrile, followed by the addition of 1.0 g of PEO (Mw = 60,000). The mixture was stirred for 12 hours to obtain a transparent solution, which was then dried in a vacuum oven at 80 °C for 12 hours to yield the polymer electrolyte membrane. The solvent-free polymer electrolyte membrane was prepared via a dry (solvent-free) synthesis method.

2. Procedure and Protocol of Online Electrochemical Mass Spectrometry (OEMS)

For OEMS analysis, 14 mm diameter $LiCoO_2$ (LCO) cathodes were prepared to maximize the signal intensity of evolved gases. The electrode slurry was formulated by mixing 80 wt.% LCO powder, 10 wt.% conductive carbon (Super P), and 10 wt.% polyvinylidene fluoride (PVDF) binder in N-methyl-2-pyrrolidone (NMP). The resulting slurry was coated onto hydrophobic carbon paper, dried at 80 °C in air, and further dried under dynamic vacuum at 110 °C for 12 hours. The final LCO cathode had an areal loading of approximately 9–11 $mg \cdot cm^{-2}$.

The OEMS cell was assembled using a 14 mm diameter LCO cathode, a 19 mm diameter PEO-based polymer electrolyte membrane, and a 16 mm diameter lithium metal counter electrode (0.5 mm thickness). The assembled cell was then promptly integrated into a state-of-the-art OEMS gas analysis system (as previously reported) and connected to the battery tester (CT-4008T-5V10mA-164, NEWARE) at 60 °C.

Gas evolution was monitored using a mass spectrometer (Hiden HPR-20 R&D) under a continuous argon flow of 3 mL/min. To ensure a stable baseline, the OEMS cell was sealed in the system and purged with Ar for 2 hours prior to electrochemical cycling. During testing, signal acquisition was performed in both scan mode (m/z = 1–200, excluding m/z = 40 and m/z = 20 to avoid saturation from argon) and multiple ion detection (MID) mode (m/z = 2, 28, 32, 44) for targeted gas analysis. In this study, fragment ions at m/z = 2, 32, and 44 were assigned

to H_2, O_2, and CO_2, respectively.

Quantification was performed by first calibrating the mass spectrometer response using certified standard gas mixtures (100 ppm H_2, CO_2, O_2, and C_2H_4 in Ar). The baseline-corrected ion signals of the evolved gases were then converted to concentrations (in ppm) by referencing the corresponding responses of the standard gases. Subsequently, the gas concentrations were translated into molar quantities using the ideal gas law (PV = nRT) and the known carrier gas flow rate (3.0 mL/min), according to the following relevant formulas:

$$k = \frac{I_{std}}{C_{std}}$$

Where C_{std} is the known concentration of the calibration gas (100 ppm), and I_{std} is the corresponding ion current (in amperes). This response factor was used to convert the measured ion current of a sample gas (I_{sample}) into a concentration (C_{sample}):

$$C_{sample} = \frac{I_{sample}}{k}$$

The obtained gas concentration (in ppm) was further converted into the total number of moles (n) released over the course of the experiment using the ideal gas law:

$$V_{sample} = \frac{C_{sample}}{10^6} * V' * \frac{P}{RT}$$

Where, V' is the carrier gas flow rate (3.0 mL/min), P is the ambient pressure (assumed to be 1 atm unless otherwise specified), T is the temperature in Kelvin, and R is the ideal gas constant (8.314 $J \cdot mol^{-1} K^{-1}$).

3. Online electrochemical GC-MS and Online Pyrolysis GC-MS Test

In the online electrochemical GC-MS test, the cells, connected to a four-way valve (in the closed state), were rapidly integrated into the NEWARE battery testing system (CT-4008T-5V6A-164, suitable for pouch-type cells; Shenzhen, China) and tested at 60 °C. Gas evolution was monitored using a gas chromatography–mass spectrometer (GC-MS, Shimadzu GCMS-

TQ8040 NX) with a continuous helium flow of 2 mL/min. To ensure a stable background, the cell was sealed within the GC-MS system and purged with helium for 2 hours prior to testing.

In the online pyrolysis GC-MS test, the sample was placed in a custom-built, temperature-programmed heating unit. To eliminate background interference, the sample was enclosed in the reaction chamber and purged with helium for 2 hours.

The GC-MS system was operated in Q3 SCAN mode over an m/z range of 5–200, with a scan rate of 0.3 s per scan. An Rt-Q-Bond (PLOT, 100% divinylbenzene) GC column (30 m length, 10 μm particle size) was used, and the column flow rate was set to 2.0 mL/min. The temperature program for all measurements consisted of three stages: (1) holding at 35 °C for 2.25 min; (2) ramping to 250 °C at a rate of 20 °C/min and holding for 2.5 min; (3) ramping to 270 °C at a rate of 20 °C/min and holding for 2.0 min.

Qualitative identification of volatile species was performed by matching both the retention times and mass fragmentation patterns with entries in the NIST library. The resulting data were collected and processed using GC-MS solution software.
